# Living in a Constant State of Fear: Phenomenological Study on Experiences of Women with High-Risk Pregnancy Waiting for Childbirth in Mpumalanga Province, South Africa

**DOI:** 10.3390/ijerph22020192

**Published:** 2025-01-29

**Authors:** Zodwa Joyce Mphego, Mathildah Mpata Mokgatle, Sphiwe Madiba

**Affiliations:** 1 Department of Public Health, School of Health Care Sciences, Sefako Makgatho Health Sciences University, Pretoria 0208, South Africa; 201812006@swave.smu.ac.za; 2 Independent Researcher, Pretoria 0182, South Africa; sphiwe.madiba@gmail.com

**Keywords:** high-risk pregnancy, experiences, psychosocial needs, emotions, support, South Africa

## Abstract

Research and obstetric medicine often ignore the emotional aspect of high-risk pregnant women in clinical management and care. Even more so, research does not adequately address psychosocial well-being in high-risk pregnancies, particularly in low and middle-income countries, including South Africa. Thus, this study aimed to explore and describe the experiences and psychosocial support needs of women waiting for childbirth in high-risk pregnancies. We conducted a descriptive phenomenological inquiry in special clinics in two hospitals in the Nkangala District Municipality of Mpumalanga Province, South Africa. A purposive sampling strategy was used to select 15 women aged 22 to 43 years, and in-depth interviews were conducted. An inductive thematic analysis approach was used. The women experience high levels of negative emotions such as shock and disbelief, fear, pain and sorrow, lack of control and hopelessness, and anxiety following a diagnosis of high-risk pregnancy. Fear was the most expressed feeling by the women; their thoughts were dominated by fear, and they lived in constant fear throughout the pregnancy journey due to the unpredictable pregnancy outcomes and the potential risks in high-risk pregnancies. Overall, the negative emotions are interlinked and bonded in the experiences of women with high-risk pregnancies, are experienced throughout the journey of waiting for childbirth, and contribute to high levels of stress and anxiety. Further exploration is needed to identify effective ways to support these women.

## 1. Introduction

The World Health Organization (WHO) considers high-risk pregnancies as a major public health challenge globally since more than 20 million women are at risk of high-risk pregnancy [1]. Whereas 10–30% of pregnancies are estimated to be at risk, the magnitude of high-risk pregnancies differs across countries [2,3]. Pregnancies are classified as medical high-risk pregnancies when they have obstetric complications that emerge during pregnancy or a pre-existing medical condition that impacts the pregnancy [4,5].

In high-risk pregnancy, maternal and foetal health is compromised, and there is a potential threat to the health of the mother and the foetus and a significant risk for maternal or foetal death [6,7]. According to WHO estimates, high-risk pregnancies constitute more than half of maternal and neonatal mortalities, and about 94% of all maternal deaths occur in low- and middle-income countries [1].

Although the global prevalence of high-risk pregnancy is estimated at 22% [7,8], the emotional aspect of a high-risk pregnant woman remains neglected in the management and care of high-risk pregnancy. Normally, pregnancy is a stressful period for women due to the physiological changes that occur and the fears, concerns, and worries about pregnancy that can lead to anxiety [9,10,11]. High-risk pregnancy evokes a range of negative emotions and psychological experiences which introduce additional stress, anxiety, and uncertainty to women with high-risk pregnancies [7,8,12].

Over the decades, obstetric medicine has ignored the emotional aspects and experiences of pregnant women with high-risk pregnancies [13,14]; thus, very few studies have investigated the range of negative emotions and psychological experiences [8,12,15]. Even more so, few studies address the emotional well-being of high-risk pregnancies [16]. This has led to a lack of locally relevant, feasible, and effective psychosocial interventions for women with high-risk pregnancies [17].

The few studies that have reported on the medical aspects and emotional experiences of women established a negative relationship between the medical aspects and emotional challenges during a woman’s high-risk pregnancy journey. However, not enough is known about their experiences of the waiting time [18,19], particularly in South Africa and other countries in sub-Saharan Africa. There is evidence from a few earlier studies conducted in developed countries of clinical relationships between the medical aspects and emotional challenges during a woman’s high-risk pregnancy journey [13]. High-risk pregnancy may predispose women to the emergence of medical conditions, pregnancy loss, and anxiety. Other studies show that the experience of waiting for childbirth puts a woman under tremendous pressure that exposes them to negative emotions such as shock, grief, fear, worry, guilt, and high levels of stress [18,20,21].

However, there is a dearth of studies conducted in South Africa, which suggests that in South Africa, our understanding of the experiences of women with high-risk pregnancies is informed by a limited body of knowledge from studies conducted elsewhere. Therefore, this study sought to explore and describe the experiences and psychosocial needs of women with high-risk pregnancies. Nurses and midwives need to understand the emotional and psychosocial needs of high-risk pregnant women to provide appropriate care and support as they wait for childbirth. Understanding the needs of women with high-risk conditions is essential to enhance the quality of maternal and childcare services and improve pregnancy outcomes.

## 2. Materials and Methods

This was a descriptive phenomenological inquiry conducted in two hospitals in the Nkangala District Municipality of Mpumalanga Province, South Africa. Phenomenology is a strategy of inquiry in which the researcher identifies the essence of human experiences about a phenomenon as described by the people involved [22,23]. Phenomenology was best suited to examine the research problem in the current study, which aimed to explore and understand the experiences of women with high-risk pregnancies waiting for childbirth. The researcher moved from the premise that the basic purpose of phenomenology is to reduce individual experiences to a description of a phenomenon [24].

### 2.1. Study Setting

The study setting was special or high-risk clinics of Middelburg Hospital, a Level 1 hospital or District Hospital, and Witbank Hospital, a Level 3 hospital or tertiary/academic hospital. Level 1 hospitals receive referrals from primary healthcare and community healthcare facilities. Level 1 hospitals refer patients to Level 2 or Level 3 hospitals for specialised care. In Middelburg Hospital, the high-risk clinic is provided once a week, whereas in Witbank Hospital, the clinic is provided twice a week. The municipality has 25 health facilities, of which only five render 24 h services. High-risk pregnancies are referred to the two hospitals where antenatal care for low- and intermediate-risk pregnant women, as well as immediate management of neonatal and obstetric emergencies, are provided. High-risk cases that need specialised supervision of care are referred from the Level 1 hospital to Level 3 for further management.

The clinics see about 80 women with high-risk pregnancies per week, and there are about 300 deliveries per month at the Middleburg Hospital and 390 at Witbank Hospital. High-risk pregnancies form part of the delivery statistics and are not distinctly separated from normal deliveries.

### 2.2. Study Sample

Participants consisted of women diagnosed with pregnancy complications that put them under a classification of high-risk pregnancy. Pregnant women were selected for participation by purposive sampling, which is the selection of participants or sources of data based on their anticipated richness and relevance of information about the phenomenon that is being investigated [25,26]. In the current study, the selection of participants was based on their experiences of the phenomenon of waiting for childbirth in high-risk pregnancies. The participants were recruited with the assistance of midwives in high-risk clinics and wards for those who were hospitalised. The midwives received all the information about this study to identify participants who met the inclusion criteria. Those who were willing to participate were referred to the interviewer. The interviewer also employed maximum variation sampling to ensure that the sample reflects a diverse group of participants diagnosed with high-risk pregnancies.

### 2.3. Data Collection

Data were collected in a quiet, private area in the high-risk clinic or ward for hospitalisation to create an environment that is conducive to free expression and to ensure privacy. The lead investigator (ZJM) conducted single and multiple in-depth conversational interviews in line with the phenomenological approach. As per the tradition of phenomenological inquiry [27], the researcher asked three broad, open-ended questions that allowed the respondents to have sufficient opportunity to express their viewpoints [28]: (1) What was it like to learn about your condition when you were first diagnosed? (2). What is it like to wait for childbirth in this condition? and (3) How has the condition and waiting for the birth of your baby affected your life? In addition, the interviewer asked an opening question to develop rapport and to put the participants at ease. These questions assisted the interviewer in exploring and developing an understanding of the phenomenon and developing a conversation on the descriptions of their lived experiences of waiting for childbirth. The interviewer also made use of probes and follow-up questions to engage intensely with the participant [29].

In purposeful sampling, the number of participants is relatively small to allow the researcher to follow participants intensely [24]. In addition, in phenomenology, a small number of participants are studied through extensive and prolonged engagement to develop patterns and relationships of meaning [30,31]. The sample size in the current study was 15 participants, and each interview took 60 to 90 min. Although the concept of data saturation does not necessarily influence data collection in phenomenology [32], the researcher was guided by the intensity of the interviews and data saturation.

The interviewer used an interview schedule developed in English and translated into IsiZulu to conduct the interviews after the participants had concluded their consultation. The interviews were conducted in IsiZulu and Sepedi, the language of the participants, to allow them to speak freely about their experiences. All interviews were conducted after written informed consent had been obtained from the women. Each interview was audio recorded with consent from the participants and lasted for about 60–90 min, in line with intense phenomenological conversational interviews [22].

Biographic data, including age, marital status, and obstetric data, were collected at the beginning of the interview to have a clear picture of the participant’s background, especially on the part of the obstetric history.

The investigators made provision for a snack and a drink in anticipation of the intensity and the length of the interview.

This study excluded pregnant women who were critically ill at the time of data collection, were below the age of 18 years, had a pre-existing psychiatric condition, were receiving psychiatric treatment, and were not willing to consent to participate.

### 2.4. Data Analysis

Qualitative thematic inductive analysis was the approach used to analyse the data, using NVivo 10. The audiotaped interviews were transcribed verbatim by a trained and experienced transcriptionist in the language of the interviews. The lead investigator who conducted the interviews translated the transcripts into English and prepared the transcripts for analysis. The investigators independently read the transcripts and listened to the audio recording to ensure that the transcripts were accurate and reflected the responses of the participants. This ensured that the investigators achieved familiarity with the data.

In line with the tradition of phenomenological inquiry, an inductive thematic analysis approach was used [33]. The first step in the phenomenological analysis is the process of reflexivity or bracketing, where the investigators set aside all presuppositions or any preconceived ideas about the phenomenon under investigation to ensure the credibility of data analysis and interpretation [34].

The investigators read the transcripts several times to familiarise themselves with the data, search for the meaning of the lived experiences of the participants, and identify initial emergent codes. This was the second step of data analysis, which yielded several initial codes. This was followed by the investigators extracting and organising the identified units’ meanings from the transcripts. The investigators met several times during the analysis process to search for themes, review, define, and name emerging themes and subthemes, and develop a coding frame. They continued to reconcile the emerging codes and themes until deep and rich themes and subthemes that described the experiences of the women had been achieved. Quotations and excerpts from the participants were used to support the themes. NVivo 12 (Lumivero’s, Burlington, MA, USA), a qualitative data analysis software, was used for the analysis process.

In this study, rigour was obtained through several techniques, and trustworthiness was ensured through the principles of credibility, confirmability, dependability, and transferability. The lead investigator practised reflexivity throughout the research process to ensure the credibility of the results by reducing researcher bias and influence on the data [35,36] as a specialist midwife who is also a facilitator of midwifery training and engages with pregnant women in this role. Reflexivity is an integral part of phenomenological research to ensure the transparency and quality of qualitative research [33,37]. The lead investigator is a nurse educator with extensive experience in midwifery. She bracketed all her experience and kept a reflexive journal to note all the processes of this research to avoid her views, preferences, values, perspectives, and position influencing the research. Interviews were conducted in the language of the participants, and the audio data were transcribed verbatim to enhance the credibility and dependability of the data. Confirmability was achieved using field notes and audit trails. To ensure that the interpretations of the data were free from investigator bias, all the authors were involved in analysis and interpretation. In addition, NVivo qualitative software was used to analyse the data [38].

### 2.5. Ethical Considerations

The Research Ethics Committee of the Sefako Makgatho Health Sciences University (SMUREC/H/311/2020: PG) provided ethical clearance for this study. The hospital authorities granted permission to conduct this study. All the pregnant women provided written informed consent before the interviews. The women were informed that their participation was voluntary and were told about the confidentiality of this study. To protect the identities of the mothers, when direct quotes are used, the name of the participants is not used as part of the descriptors that provide context for the findings.

## 3. Results

### 3.1. Demographic Profiles of Participants

The sample included 15 high-risk pregnant women recruited from high-risk clinics. Their ages ranged from 22 to 43 years, with a mean age of 31 years. Seven (7) of the women were single, seven (7) were married, and one (1) was divorced and in a new relationship. With regards to employment status, six (6) women reported that they were unemployed, and nine (9) were employed before the diagnosis of high-risk pregnancy. Half of the participants had completed high school education, five (4) had tertiary education, and three (3) had less than high school education.

Seven (7) women described their pregnancy as unplanned, and eight (8) as planned pregnancy. Two (2) participants were primigravida, one (1) of them was an advanced maternal age, and four (4) women had no live children. Of the women with no live children, one was a gravida 7 with recurrent miscarriages, and the rest of the participants had at least one live child, a range of 1 to 3 live children (Table 1).

### 3.2. High-Risk Conditions of Participants

The participants in this study had several co-occurring conditions that increased their high-risk conditions. Pregnancy-induced hypertension (PIH) was the highest high-risk condition and occurred with other high-risk conditions. Four women were diagnosed with PIH only, one had PIH and asthma, one had PIH and gestational diabetes (GD), and one had PIH and miscarriages. Miscarriage was the second-highest high-risk condition with some underlying conditions like diabetes and PIH, 10 of the women had previous miscarriages, one outstanding woman had seven unexplained miscarriages, and the rest of the women had between one and five miscarriages occurring with other conditions like asthma and a BMI of 40. One woman had GD, one had an incompetent cervix, one of them had a premature rupture of membrane (PROM), and one was in advanced maternal age (Table 2).

### 3.3. Themes

Five main themes and 12 supporting subthemes that explain the experiences of waiting for childbirth in high-risk pregnancies emerge from the data analysis (Table 3).

#### 3.3.1. Views of the High-Risk Clinic

The interviews explored how women experienced attending high-risk clinics. The data revealed that some of them found it strenuous to attend high-risk clinics, while others viewed their attendance as a means to understand their condition and get assisted about their condition. Views about their attendance were expressed as stressful experiences, getting assistance with high-risk conditions, and the formation of social networks.

##### Stressful Experience

Participants expressed that it was stressful to attend high-risk clinics weekly or bi-weekly. They also stated that attending the high-risk clinic was financially demanding since they did not anticipate high-risk pregnancies that put a demand on them.

*It makes me nervous… Sometimes it makes me emotional. The issue of coming here every week makes me feel that something is wrong with me because if it was normal, I would visit the clinic monthly. So, it makes me emotional at times. It’s a challenge to have a high risk because you don’t rest; you have to go to the hospital now and then so that they can check if everything is fine (32 years old gravida 3 para 1, had a miscarriage with twins, currently carrying triplets and has anaemia).*

*It is very stressful because you must attend twice, you go to the normal clinic and high-risk clinic, and you must wake up early. It is very stressful. Sometimes you can’t go to some of your commitments (36-year-old gravida 5 para 1 with three miscarriages, one live premature at 7 months, and PIH).*

*Coming here is expensive; it involves petrol and money. We come here and stay for a long time, and you have to buy food (32-year-old gravida 4 para 2 with one miscarriage).*

Some of the pregnant women realised that being at the high-risk clinic indicated the seriousness and abnormality of the condition. It gave women a better understanding of high-risk conditions.

*I am attending and getting help. I know that I will get help if I need it. They check thoroughly. I feel that I am safe because I am attending (43-year-old gravida 1 para 0, AMA in a primigravida).*

*“Coming here for me, it shows that something is not normal, so it means this thing [pregnancy-induced hypertension] is serious, and it shouldn’t be like this.” I am starting to understand that this condition is dangerous (30-year-old gravida 1 para 0 with PIH).*

##### Getting Assistance

On the other hand, those who have attended for long felt that being at the high-risk clinic was helpful and that without attendance at the high-risk clinic, they would have already lost their babies.

*The lives of the mother and the baby are in danger. That’s why I say it is important to attend high-risk clinics. What I understand is that they help a lot; it must be done. Yes, it must be there to protect the mother and the child because if you just sit at home, you don’t know anything about your situation (34-year-old gravida 4 para 3 with asthma, DM, and PIH).*

*The main reason for coming here is that they want to save the mother and baby (32-year-old gravida 4 para 2 with one miscarriage).*

*The sister does what she must do. She helps me, puts me on the scan, checks, then gives you treatment. If they didn’t help me by stitching the cervix as they said, my womb would let go. It could have let go of the baby before seven months (28-year-old gravida 9 para 3 with five miscarriages, DM, and BMI of over 40).*

##### Establishing Social Networks

Women felt that attending high-risk clinics provides them with an opportunity to connect with other women who have challenges and conditions similar to theirs. The conversations held while waiting to be seen at high-risk clinics were found to be helpful and made some of them change their perceptions about a high-risk pregnancy and the outcome thereof.

*The more we talk as high-risk patients, the more you heal because everyone comes with their challenges. Then it makes you feel better, and it makes you have hope that it will all pass because others have undergone the same situation and managed to pull through. I will also overcome even though it’s tough. The conversations we have here at a high-risk clinic help us to persevere (gravida 4 para 2 withone miscarriage and one premature and incompetent cervix).*

#### 3.3.2. Understanding of High-Risk Condition

The women expressed frustrations because they did not receive adequate information from the nurses and doctors both at the high-risk clinic and hospital regarding their conditions. While they recalled being diagnosed with high-risk conditions, they were unable to comprehend the information communicated to them. Two themes that explain this theme emerged.

##### Lack of Comprehensive Understanding

The women lacked sufficient knowledge and understanding about high-risk conditions; the understanding of high-risk conditions was limited to just the basics. The poor understanding of the high-risk condition made them worry and not to be able to explain the condition to significant others, especially their spouses and family members.

*I didn’t have enough information; I was told I had high blood pressure, but I did not know what the condition was (22-year-old gravida 2 para 0 with chronic HP).*

*I don’t think I have enough information; I keep asking the sisters at the clinic about this high blood pressure. I never understood, and I kept asking, but I never got the correct answer. I don’t want to lie to you (36-year-old gravida 5 para 1 with three miscarriages, one live premature at 7 months, and PIH).*

*The information I got is not full because even if they can ask me, I will not be able to explain in full what high-risk is. All I know is just the basics: I might have complications, and I should eat properly, avoid fatty foods, and reduce salt intake (30-year-old gravida 1 para 0 with PIH).*

*I just told them that I am going to have a baby at an advanced age. I don’t have much information, and my family doesn’t understand. They are just like me (43-year-old gravida 1 para 0, AMA in a primigravida).*

##### Understanding Improved over Time

For women who were hospitalised, regular attendance at the high-risk clinic and exposure to other women with similar conditions improved their understanding of the high-risk condition.

*At that time, I told them whatever they told me. I just told them what the doctor said. I did not have enough knowledge to explain, but as time went on during my stay here in the hospital, I started understanding, and I saw others also admitted with the same condition. Then I realised that I am not the only one with the condition (32 year-old gravida 3 para 2 with PROM).*

#### 3.3.3. Emotional Response to High-Risk Condition

The data revealed that women with high-risk pregnancies experienced a range of emotional issues following the diagnosis of high-risk pregnancy. The most dominant emotions the women experienced were disbelief and shock, anxiety, stress, hopelessness, sorrow, and fear.

##### Disbelief and Shock

Disbelief, shock, and confusion were the emotions that were related strongly to the women’s experience at the diagnosis of the high-risk condition. Initially, the women had difficulty understanding and internalising the idea that they were suffering from a high-risk pregnancy.

*I don’t understand a thing. All I know is that my blood pressure is high. I knew high blood to be a disease of old people, so I was shocked when I was told I have high blood I was really shocked at the diagnosis of high blood at my age (36-year-old gravida 5 para 1 with three miscarriages, one live premature at 7 months and PIH).*

*I got very confused and frustrated a lot (34-year-old gravida 4 para 3 with diabetes, asthma, and HP).*

*My main concern is that I don’t understand at my age. Am I too old, or what is going on? Or was I too fast to have babies, or was this condition inherited from my family or what? I don’t know really (28-year-old gravida 4 para 1 with two miscarriages).*

Initially, disbelief and poor understanding of the condition led to poor acceptance of the diagnosis of high-risk pregnancy. Their narratives indicated that they had many unanswered questions about the diagnosis in their minds. Their knowledge of certain conditions like HP was that it was a condition of old people. They, therefore, could not reconcile being pregnant and having HP.

*I know it to be a disease of old people. Eish… I am not ok. I am shocked. “What is this?” How come I have high blood pressure? I don’t feel ok because sometimes I’m confused and ask myself why me. Why do other people continue as normal without high blood? (36-year-old gravida 5 para 1 with three miscarriages, one live premature at 7 months, and PIH).*

*I fear that I can’t accept this condition. As I am waiting for results, I keep thinking that they will tell me I can’t carry this baby for 9 months, maybe I will lose the baby or die. That is my biggest fear (36-year-old gravida 3 para 2 with chronic DM and PIH).*

##### Pain and Sorrow

Participants experienced intense pain and sadness as a result of previous loss in another pregnancy or pregnancies, as some of the women had recurrent miscarriages. The feeling of sadness resulted from the uncertainty of pregnancy outcomes following the diagnosis of high-risk conditions.

*Eish… it’s painful to lose babies. It’s not nice not to have your photocopy [own child]; it’s painful. At times, you feel you are not like the other women (34-year-old gravida 8 para 0 with unexplained miscarriages).*

*Joh… It’s so painful; I don’t want to lie to you. You always look back at what happened with the previous pregnancy. You are forever scared. The miscarriages are a concern. You are never free until you see the baby (36-year-old gravida 5 Para 1 with three miscarriages, one live premature at 7 months, and PIH).*

The narratives also revealed that women were scared to connect with the baby because of the fear that after they connected, then the baby might die. They felt that connecting with the baby would result in more pain if the baby died.

*I feel very sad. It’s very painful because you connect with the baby, then the next thing, the baby dies, or I die; it’s very painful (34-year-old gravida 4 para 3 with DM, HP, and asthma).*

*You also wish to buy pink clothes, but on the other hand, you ask yourself a question: what if you buy clothes and you lose the baby? The clothes will hurt me if I lose the baby (22-year-old gravida 2 para 0, lost first baby to HP).*

The women also reported that their partners also experienced pain and sorrow on hearing about the high-risk condition.

*I saw the pain even though he didn’t say it, but the pain of your partner, you see it. Even if he hides it, you can see he is in pain (28-year-old gravida 9 para 3 with five miscarriages, DM, and BMI of over 40).*

##### Loss of Control and Hopelessness

As women with high-risk pregnancies wait for the birth of the baby, one of their biggest fears was to lose the baby. They expressed feelings of hopelessness and desired to have some level of control over the situation. Feelings of an inability to control the condition heightened their anxiety, which they expressed about themselves and their unborn babies.

*When it comes to high-risk pregnancy, it’s either good or bad results; it is very difficult, and there’s nothing you can do. Eish… It’s difficult because you don’t know what they are going to tell you. Maybe you will find that the baby is not ok, or you will come across some difficulty. You keep having negative thoughts. They keep coming to your mind. I wish I could have control of the situation (34-year-old gravida 8, para 0 with unexplained miscarriages).*

*It’s tough because sometimes I want to give up. Telling myself that maybe this one will be like the others [previous miscarriages]. I might also lose him (36-year-old gravida 4, para 2 with one miscarriage and one premature due to incompetent cervix).*

*So, what if I die? That’s my fear… I’m scared that maybe I may lose these babies. That’s why sometimes I just leave things as they are (32-year-old gravida 3, para 1, had a miscarriage with twins, currently carrying triplets, and has anaemia).*

#### 3.3.4. A Constant State of Fear

The narratives of the women indicated that they experienced fear of what they had heard about a high-risk pregnancy. They were terrified at the diagnosis of a high-risk pregnancy and expressed fears of losing the baby, of the outcome of test results, of death of self, and of premature delivery while they waited anxiously for childbirth. A poor understanding of the high-risk condition worsened the fear. Three subthemes highlight fear’s impact on women while they await childbirth.

##### Fear of the Outcome of Test Results

Women with high-risk pregnancies undergo numerous procedures and tests to monitor the progression of the high-risk condition, growth, and well-being of the foetus and its effect on the condition of both the mother and the baby. They expressed fear of various test and procedure results that are part of the management and care of high-risk pregnancies. They described how they waited for results with fear of negative outcomes or worsening of the condition and worried about the implications of the results.

*It was very difficult because as you wait for the results, you ask yourself questions. Are they going to be positive or negative? So, the more you wait, the more frustrated you become (36-year-old gravida 4 para 2 with one miscarriage and one premature due to incompetent cervix).*

*I keep asking them how my results are. For me, it’s a good thing because I want to know what my status is, how dangerous the sugar levels, and what damage is it doing to the baby, and how is the baby (36-year-old gravida 3 para 2 with chronic DM and PIH).*

*I was anxious to see whether it had dropped or if it was still high. What if it is highWhat is going to happen to me? It scares me if the BP is high (30-year-old gravida 1 para 0 with PIH).*

##### Fear of Death of Self or Baby

Women expressed fear about the unborn baby’s survival and health. The unexpected diagnosis of a high-risk pregnancy created feelings of worry and fear of the potential loss of the baby and imminent death of self or the baby. However, for most women, the strongest fear they reported was fear of death of the baby.

*I keep thinking that they will tell me I can’t carry this baby for 9 months, maybe I lose the baby or die. That is my biggest fear (36-year-old gravida 3 para 2 with chronic DM and PIH).*

*My biggest fear is to lose the baby. A miscarriage is very painful (34-year-old gravida 8 para 0 with unexplained miscarriages).*

*What I understand is that it is a killer because when the heart starts beating fast, you become short of breath, and this can kill you, so it’s easy to die of high BP (22-year-old gravida 2 Para 0 lost first baby to HP).*

*I was scared because, at times, they say the baby’s heartbeat is slow; they say the baby does not get enough oxygen. So, it was very scary…. Yes, I was scared that the results would come back saying the oxygen level was low and that put my baby in danger, or it could put me in danger because it could also kill me (22-year-old gravida 2 para 0 who lost baby to HP).*

The lack of comprehensive understanding and knowledge of high-risk conditions contributed to the fear that women with high-risk pregnancies experienced.

*It terrifies me because I hear stories that when you are at high risk, there is a possibility that they can take the baby out before time. Another thing is that if you don’t take care of yourself, there’s a possibility that you can put your baby in danger (30-year-old gravida 1 para 0 with PIH).*

*I was scared. I thought about me and my baby. It wouldn’t be nice to have diabetes while expecting a baby. I knew I didn’t have diabetes, but I was scared that if I had it because some things just happen out of the blue (43-year-old gravida 1 para 0, AMA in a primigravida).*

##### Fear of Another Miscarriage

One of the biggest fears of women who had previous miscarriages was having another miscarriage. Thus, they lived in constant fear, and each time they had mild pain, they thought that it was another miscarriage.

*Eish… I thought about the miscarriage, and the issue of miscarriages came to mind. You always look back at what happened with the previous pregnancy (36-year-old gravida 5 para1 with three miscarriages and PIH).*

*Joh… It’s so painful; I don’t want to lie to you. You are forever scared. The miscarriages are a concern you are never free of until you see the baby (36-year-old gravida 5 para 1 with three miscarriages, one live premature at 7 months, and PIH).*

*It was very difficult to talk or tell them [family] because of my previous miscarriages, and we are all [family] scared that I might get another miscarriage (28-year-old gravida 4 para 1 with two miscarriages).*

*Sometimes, I get scared that it might happen again that I get a miscarriage (36-year-old gravida 4 para 2 with one miscarriage and one premature due to incompetent cervix).*

#### 3.3.5. Support Needs While Waiting for Childbirth

Data revealed that women need emotional support, financial support, help with household chores, and assistance with taking care of their children from partners, family, and other significant people in their lives, such as friends, while waiting for childbirth.

##### Emotional Support

Women received emotional support from their partners to cope with waiting for childbirth while also managing the high-risk condition. Being supported by their partners assisted them to deal better with the high-risk conditions.

*The support I received from my partner and family made me cope with the high-risk condition; it made it easy for me to deal with the condition. Above all, my partner encouraged me to wait, and that made me persevere (32-year-old gravida 3 para 2 with PROM).*

*My partner is very supportive; he gives me love, and he keeps calling me when he is at work and asking me how many times the baby has kicked. He says I am with you no matter what, I am really not alone in this situation, and they also stopped us to have sex, but he never demands sex (34-year-old gravida 8 para 0 with unexplained miscarriages).*

A few women felt that the high-risk condition made their relationships with their partners stronger.

*The high-risk condition has brought us closer than before; he is far away, but he calls more than once per day to find out how am I doing. I am also dependent on him financially (32-year-old para 1 gravida 3 with one miscarriage of twins and now carrying triplets).*

*My partner is very supportive and tolerant. We were told not to have sex because of the stitch, and he perseveres and guides me, telling me what to do for the sake of the baby. We have been waiting for too long, so we always talk about the pregnancy and how to deal with it to save the baby. I think waiting for the baby has made our relationship stronger (32-year-old gravida 4 para 2 with one miscarriage).*

##### Support with Household Chores

The management of high-risk pregnancy includes regular monitoring at a specialised clinic, strict bed rest at home, or hospitalisation for a prolonged period. Strict bed rest restricted the women from doing some duties, and they reported that their partners carried out most of the household duties.

*The partner is responsible for household chores, and he has even stopped me from heavy duties, which makes me feel special even though I am facing this challenge (32-year-old gravida 4 para 2 with one miscarriage).*

*While in the hospital, my partner does all the household duties, and that makes me feel better to know that I did not leave my kids in the street. They are at least with a responsible person (para 2 gravida 3 with PROM).*

On the other hand, for women who did not live with their partners, their narratives showed that they received support from family members and, to a lesser extent, friends.

*I moved to live with my sister, and she does all the duties because I am on bed rest (34-year-old gravida 8 para 0 with unexplained miscarriages).*

*My family understands, and they are supportive because they know this thing of high blood. I can rely on my mother for information (22-year-old gravida 2 para 0 with chronic hypertension).*

##### Financial Support

Besides the concerns about the high-risk condition, women also experienced financial strains. Those who were employed at the time of the high-risk diagnosis had to stop working, while some were unemployed and depended on the child support grant that they received for the other children. The women reported that they also received some form of financial support from partners and family.

*My partner does help financially because we only survive with a child support grant, and this hospital is very far. You spend R200 return trip (34-year-old gravida 4 para 3 with DM, asthma, and HP).*

*I depend on my grandmother for financial support, but my partner does help out (28-year-old gravida 4 para 1 with two miscarriages).*

## 4. Discussion

The purpose of this research was to explore and understand the experiences of women diagnosed with high-risk conditions as they wait for childbirth. This study was conducted within a context where women attended high-risk clinics regularly for monitoring of high-risk pregnancy and foetal growth and well-being. This research revealed that their initial experiences of attending high-risk clinics were stressful, and they lacked an understanding of their high-risk conditions when they were first diagnosed. However, they gained knowledge and information about high-risk conditions through regular attendance at the high-risk clinics and engaging with other women with high-risk conditions. This study further found that a lack of knowledge and understanding of high-risk conditions was the fundamental cause of the worries that women had about high-risk pregnancy. Prior research reported similar findings [39]. Feelings such as fear and anxiety can be alleviated when women have adequate information about the diagnosis [11]. Therefore, midwives are in a unique position to assist women with high-risk pregnancies by providing easily translated medical terms and emotional support [40].

We found that a diagnosis of high-risk pregnancy evoked a range of emotions due to the difficulty in dealing with the high-risk diagnosis. The women in this study experienced high levels of negative emotions such as shock and disbelief, fear, pain and sorrow, lack of control and hopelessness, and anxiety. The negative emotions were due to the stress of dealing with the high-risk pregnancy, the potential risks involved, and the unpredictable pregnancy outcomes. Our findings are consistent with what has been reported in previous studies [13,41].

This study found that when women were first diagnosed with high-risk conditions, their first response was shock and disbelief; however, as time progressed, they experienced intense pain and sorrow in various degrees. The feelings of sorrow and sadness were very intense among women who had experienced recurrent miscarriages and stillbirths. The findings are similar to what has been reported in previous studies [11,42,43]. Most of the women started to grieve for the loss of the baby at the diagnosis of the high-risk condition. The grieving for the potential loss of the baby resulted in fears of connecting with the baby who may die. They feared that bonding with the baby would result in more pain and sorrow if the baby died. Similar findings were reported in previous studies [13,40,44]

As women with high-risk pregnancies wait for the birth of the baby, one of their biggest fears is the potential loss of the baby. Loss of control over the high-risk condition and pregnancy outcomes also evoked emotions of frustration, powerlessness, loneliness, and social isolation, particularly during hospitalisation and prolonged bed rest. Hospitalisation abruptly created feelings of loss of control over their home routines, care of children, self-care, and meeting their own needs. Women in prior studies also felt frustrated and anxious by the inability to control their bodies due to the high-risk condition [39,45,46].

The women in this study also felt trapped in the condition and by the condition. They expressed that the high-risk condition kept their lives on hold and forced them to adjust their lives to suit the condition as they waited for childbirth. The experiences of women in this study were similar to what women experienced in another study conducted in South Africa [42] and other studies [18,39]. The study findings showed that loss of control heightened the anxiety of the women as they waited for childbirth. Research further shows that feelings of loss of control magnify the impact of stress and leave women vulnerable to stress and depression [40].

Pregnancy-related fear was a common feeling experienced and expressed by women as they waited for childbirth. They reported fear of imminent death of self and fear of potential loss of the baby. Yet, fear of death of the unborn baby was the strongest reported fear by women, particularly those who have had a previous loss of a baby to a high-risk condition. They lived in constant fear throughout the journey of waiting for childbirth as the thought of another miscarriage with every mild pain dominated their feelings. Other studies documented similar findings of thoughts that are dominated by fear after a high-risk diagnosis [39,40,45]. Fear in this study reflected women’s hopelessness in facing the high-risk condition. As stated, they were afraid of connecting and bonding with the baby because of the fear that the baby might die and leave them with more pain.

The findings showed that fear was a prevalent feeling related to the uncertainty and loss of control of the pregnancy outcomes. For the women in this study, fear was intensified by a lack of sufficient knowledge and understanding of the high-risk pregnancy. Thus, their perception of the high-risk conditions was that it kills. Their thoughts were dominated by fear throughout the pregnancy journey. Other studies reported on the state of constant fear that women with high-risk pregnancies experience [11,40,45]. We further found that living in constant fear of the outcome of the high-risk pregnancy increased the levels of anxiety, stress, and uncertainty, predisposing them to poor mental well-being. The study findings corroborate those of prior studies [47,48,49,50,51].

This study further established that the women had different support, which depended on the severity of the high-risk condition, prolonged hospitaliation, and bed rest at home. Strict bed rest restricted them from performing even the simplest of tasks, resulting in their dependence on others with home chores and taking care of the children. Support from significant others offers women a sense of comfort and lessens the feelings of loneliness for those who were hospitalised. Other studies reported that social support decreases stress, increases coping skills, and contributes to the psychological well-being of women with high-risk pregnancies [42,52,53].

### Study Limitations

This study was limited to a small area of Mpumalanga Province and included a small sample of women; however, the sample was diversified and included women recruited from high-risk clinics and maternity wards of two hospitals to recruit women who were hospitalised. The sample consisted of women with varying clinical and sociodemographic characteristics. Secondly, the women were purposely sampled to ensure the representation of all the clinical conditions that meet the definition of high-risk pregnancy. The diversity of the study sample allowed the researcher to capture the core shared and lived experiences of women with high-risk pregnancies.

However, women who might have expressed their experiences of waiting for childbirth in high-risk pregnancies with high-risk conditions that are not treated in the two hospitals in the district were not included in this study. For example, cardiac conditions in pregnancy are referred to and treated in academic hospitals in Gauteng Province. Nevertheless, the researcher believes that women with cardiac conditions represented a minority of the high-risk conditions and could not have biased the target population of this study.

While the investigators planned to conduct multiple interviews, only two participants could be interviewed twice, and subsequent interviews could not be conducted due to the COVID-19 pandemic lockdown. By the time the lockdown restrictions were lifted, the participants had delivered.

## 5. Conclusions

This study concludes that the emotions and feelings experienced by women with high-risk conditions while waiting for childbirth are interlinked and bonded in the experience of high-risk pregnancy. Fear became the most expressed feeling by women, and they lived in constant fear throughout the whole pregnancy due to the uncertainty of the outcome of the high-risk pregnancy. However, fear was experienced at varying levels during pregnancy; for example, fear was intensified among women who had recurring miscarriages as they feared another pregnancy loss.

Feelings such as fear and anxiety experienced during high-risk pregnancy can be alleviated when women have adequate information about the diagnosis. It is, therefore, essential that nurses and midwives consider having patient information-sharing sessions and educational programmes that can reduce the fear and stress associated with high-risk pregnancies. Educational and health promotion materials may improve the pronounced lack of knowledge about high-risk conditions that were observed in these women.

Furthermore, there is a need for midwives with adequate knowledge and skills to manage high-risk pregnant women’s emotions to improve pregnancy outcomes. The care provided to women in high-risk clinics should go beyond clinical care only and provide support, which also encompasses emotional aspects. In addition, nurses and midwives should consider introducing group sessions where women with high-risk conditions can meet, share their experiences, and support one another. The sessions will serve as their social support network to improve their coping strategies. The integration of psychosocial care in the management of high-risk pregnant women as they wait for childbirth is crucial to attaining sustainable development goal 3 to improve maternal health.

## Figures and Tables

**Table 1:** Demographic and obstetric profile of women with high-risk pregnancies.

Age	Marital Status	Educational Status	Employment Status	Gravida	Parity	Pregnancy Status	Live Children
32	Married	Tertiary	Unemployed	3	1	Planned	1
31	Single	Matric	Employed	2	1	Planned	1
31	Married	Tertiary	Employed	5	2	Planned	2
36	Married	Matric	Employed	5	1	Planned	1
22	Married	Matric	Employed	2	0	Unplanned	0
28	Single	Grade 11	Unemployed	9	3	Unplanned	3
30	Married	Tertiary	Unemployed	1	0	Planned	0
43	Single	Matric	Employed	1	0	Planned	0
32	Married	Matric	Employed	2	2	Unplanned	2
34	Divorced	Grade 11	Unemployed	4	3	Unplanned	3
32	Married	Tertiary	Employed	5	2	Unplanned	2
34	Single	Grade 11	Unemployed	7	0	Planned	0
33	Single	Matric	Employed	3	2	Unplanned	2
32	Single	Matric	Employed	4	2	Planned	2
28	Single	Tertiary	Unemployed	4	1	Unplanned	1

**Table 2:** Pregnancy related high-risk conditions of the participants.

High-Risk Condition.	Previous Complications	Gestational Age	Living with
Anaemia and multiple pregnancies	One miscarriage and multiple pregnancies	31 weeks	Partner and son
Pregnancy-induced hypertension	One miscarriage	16 weeks	Partner
Pregnancy-induced hypertension	One stillborn and one miscarriage	37 weeks	One child
Pregnancy-induced hypertension	Three miscarriages	21 weeks	Partner
Pregnancy-induced hypertension	One miscarriage and PIH	21 weeks	Partner
Chronic diabetes and BMI of 40	Five miscarriages	16 weeks	Partner
Pregnancy-induced hypertension	None	29 weeks	Parents and siblings
Advanced maternal age	None	34 weeks	Uncle
Premature rupture of membranes	Pregnancy-induced hypertension	37 weeks	Partner and children
Pregnancy-induced hypertension and asthma	None	31 weeks	Three foster children and three of hers
Incompetent cervix	Incompetent cervix and two miscarriages	29 weeks	Partner
Recurrent miscarriages	Seven miscarriages	37 weeks	Partner
Diabetes and PIH	None	32 weeks	Two children
Premature birth and miscarriage	One miscarriage	30 weeks	Partner
Recurrent miscarriages	Two miscarriages	24 weeks	Parents

**Table 3:** Summary of emergent themes.

Themes	Subthemes
Views about the high-risk clinic	Stressful experience
	Getting assistance
	Establishing social networks
Understanding of high-risk condition	Lack of comprehensive understanding
	Understanding improved over time
Emotional response to high-risk condition	Disbelief and shock
	Pain and sorrow
	Loss of control and hopelessness
A constant state of fear	Fear of the outcome of test results
	Fear of death of self or baby
	Fear of another miscarriage
Support needs	Emotional Support
	Support with household chores
	Financial support

## Data Availability

The data that support the findings of this study are available from the corresponding author upon reasonable request.
